# 

**Published:** 1893-01

**Authors:** 


### CONTENTS.

ORIGINAL COMMUNICATIONS—

TREATMENT OF TALIPES EQUINO-VARUS. By C. W. Barrier, M. D., Columbus, Ga.. 641

PLEURISY. By E. S. McKee, Cincinnati, Ohio.......... 651

UTERINE FIBROMATA. REMOVAL OF TWENTY-SEVEN WITH TWO DEATHS. By J. M. Baldy, M. D. Philadelphia.......... 656

THE TREATMENT AND MANAGEMENT OF ASTHMA. By Thos. J. Mays, M. D., Philadelphia 663

TWO CASES OF ACCIDENTAL WOUND OF THE FEMORAL ARTERY. By E. Dickson, M. D., Coal Creek, Tenn.......... 667

SOCIETY REPORT—

SOUTHERN SURGICAL AND GYNECOLOGICAL ASSOCIATION.......... 669

EDITORIAL—

SQUEAL FROM THE ECLECTICS.......... 679

SELECTIONS.......... 681

MEDICAL ITEMS.......... 697

BOOK REVIEWS.......... 701

INDEX TO ADVERTISEMENTS.......... xii

ITEMSOF INTEREST.......... x-xi

